# Sonographic measurement of ear length among normal fetuses of pregnant Igbo women in port Harcourt, Nigeria

**DOI:** 10.4314/ahs.v21i1.43

**Published:** 2021-03

**Authors:** Felicitas Idigo, Kingsley Ajibo, Angel-Mary Anakwue, Uloma Nwogu, Ebbi Robinson

**Affiliations:** 1 University of Nigeria Faculty of Health Sciences and Technology, Medical Radiography and Radiological Sciences; 2 University of Nigeria, medical radiography and radiological sciences; 3 Rivers State University Teaching Hospital, Radiology

**Keywords:** Fetal ear length, sonographic measurement, chromosomal aneuploidy

## Abstract

**Background:**

Fetal ear length measurement has been associated with some clinical values: sonographic marker for chromosomal aneuploidy and for biometric estimation of fetal gestational age.

**Objectives:**

To establish a baseline reference value for fetal ear length and to assess relationship between fetal ear length and gestational age.

**Methods:**

Ear length measurements were obtained prospectively from fetuses in 551 normal singleton pregnancies of 15 to 41 weeks gestation. Normal cases were defined as normal sonographic findings during examination plus normal infant post-delivery. The relationship between gestational age (GA) in weeks and fetal ear length (FEL) in millimeters were analyzed by simple linear regression. Correlation of FEL measurements with GA, biparietal diameter (BPD), Head circumference (HC), Abdominal Circumference (AC), Femur Length (FL) and maternal age (MA) were also obtained.

**Results:**

Linear relationships were found between FEL and GA (FEL=0.872GA-2.972). There was a high correlation between FEL and GA (r = 0.837; P = .001). Good linear relationship and strong positive correlation were demonstrated between FEL and BPD, AC, HC, and FL (p<0.05).

**Conclusion:**

The result of this study provides normal baseline reference value for FEL. The study also showed good linear relationship and good correlation between FEL and fetal biometric measurements.

## Introduction

A number of researches have been carried out to correlate fetal ear length with fetal biometric measurements and gestational age. In addition, efforts were made to evolve fetal ear length nomogram for the different ethnic populations studied^1^–^5^. Many of the studies identified the usefulness of the fetal ear length as a sonographic marker for fetal aneuploidies^1^, ^3^, ^4^.

The probability of having an aneuploid pregnancy increases with increasing maternal age^6^. These chromosomal abnormalities are major causes of prenatal death and childhood handicap^7^. The defects could be internal or external and efforts have been made over time to detect the abnormalities in utero to enable possible early intervention. Although invasive and associated with risk of miscarriage, cytologic evaluation by amniocentesis, chorionic villus sampling or fetal blood sampling are still the definitive method for antenatal identification of fetuses with abnormal number of chromosomes^7^.

Sonographic measurements and findings of shortened femur length, shortened humerus length, mild renal pyelectasis, hypoechoic bowel, duodenal atresia, and hypoplasia of the middle phalanx of the fifth digit, and congenital heart disease, in second trimester, have been proposed as ways of detecting these fetuses with abnormal number of chromosomes^8^, ^9^, ^10^, ^11^. Unfortunately none of the methods is foolproof as most of the abnormal ultrasound findings are inconsistent in abnormal chromosome fetuses.

Recent advances in ultrasound technology have improved the detection of congenital abnormalities in utero by the use of various sonographic markers at different gestational ages. Between 11–13 weeks gestational age, Nuchal Translucency (NT) measurement above 3.5mm and nasal bone hypoplasia are sonographic markers for early prediction of fetal aneuploidy^12^, ^13^, ^14^. Other markers used in this period are ductus venoses spectrum, and tricuspid regurgitation^15^.

In 11–13 weeks, Nuchal Translucency (NT) scan and nasal bone are sonographic markers for early prediction of fetal aneuploidy^12^; other markers used in this period are ductus venoses spectrum, and tricuspid regurgitation.

Measurement of fetal ear length at prenatal ultrasound, either alone or in combination with other sonographically detectable structural abnormalities, has been shown to be a sonographic marker for fetal aneuploidy^2^, as babies with these abnormalities have short ear length. Notably, abnormal small ears have been noted to be one of the most consistent clinical findings in newborn and infants with trisomy 21 and other aneuploidies^16^, ^17^, ^18^.

Fetal ear length measurement can be obtained routinely when a pregnant woman comes for routine antenatal or anomaly scan. Ultrasound is non-invasive and cheap with absence of radiation hazard, making it a modality of choice. However the aforementioned studies were carried out in western ethnic enclaves and races. There is no record of study on the measurement of fetal ear length for any Nigeria population seen in literature. It is therefore, necessary, to establish a regional derived baseline reference value for normal fetal ear length and determine a cut-off value for fetal ear length assessment during prenatal sonographic assessment in Igbo population.

It is therefore necessary to establish a regional derived baseline reference value for fetal ear length to determine the cut for fetal ear length in a Nigerian population.

## Methods

Using convenience sampling technique, fetal ear measurements were obtained in 850 women with singleton pregnancies, undergoing prenatal sonography between 15 and 40 weeks' gestation, over a period of 12months. Ethical clearance was obtained from Rivers State University Teaching Hospital Ethical Committee, while informed consent was obtained from each of the subjects before the study commenced. Confidentiality of all information obtained from the subjects was also assured. The quipment used for the study was Mindray DC-N3 ultrasound scanner, manufactured in 2014 by Shenzhen Mindray Biomedical Electronics Co, Ltd China.

The equipment used for this study was Mindray DC-N3, a product of Shenzhen Mindray Biomedical Electronics Co, Ltd China, manufactured in 2014.

Gestational age was assessed using the women's last menstrual period in conjunction with ultrasonographic estimates of biparietal diameter (BPD), head circumference (HC), abdominal circumference (AC), and femur length (FL) according to established nomograms for these parameters^19^. Exclusion criteria included cases with sonographic abnormalities such as intrauterine growth retardation, fetal structural anomalies, oligohydramnios or polyhydramnios, history of chromosomal abnormality in previous offspring(s), irregular cycles, unreliable menstrual date and known diabetics. In addition, pregnant women with discrepancy of > 14 days between the postmenstrual age and sonographic gestational age were excluded from the study. Also excluded from participating were pregnant women in whom the fetal ear length could not be measured due to positional problem.

As amniocentesis was not routinely carried out in the facility of study, post natal assessment of babies at birth was used to confirm normalcy. Thus for this study, normal cases were defined by absence of sonographic abnormalities with a normal infant examination at birth20. The final study population then comprised of 551 healthy fetuses, as 299 (35.18%) cases were lost to follow-up or poor obstetrics outcome and therefore excluded from study. A single sonographer with more than ten years' experience in obstetric ultrasound scanning carried out prenatal sonography on each participant. Although some of the participants underwent multiple prenatal sonography, only one set of the measurements obtained for each person was recorded for the purposes of this study.

**Figure 1:**
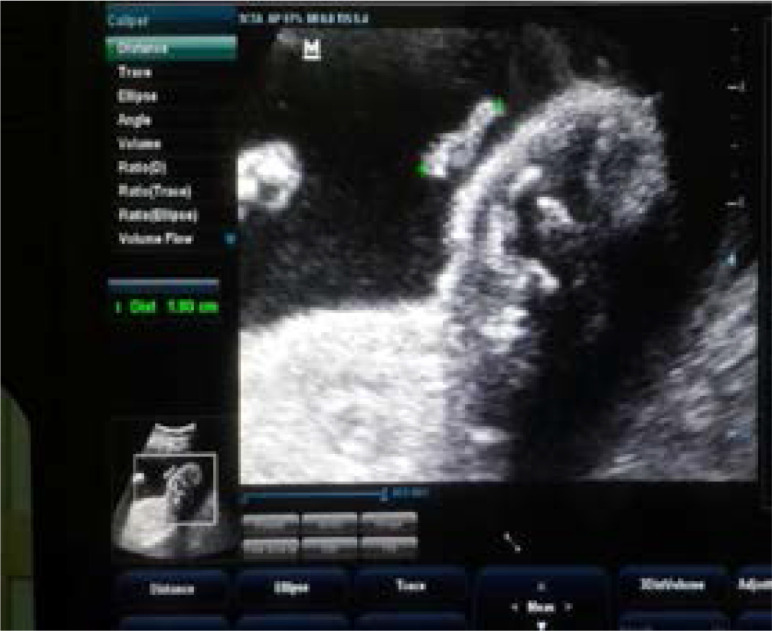
Fetal ear length taken from the tip of the helix to the end of the lobe (caliper) in coronal view at 29 weeks of gestation.

**Figure 2:**
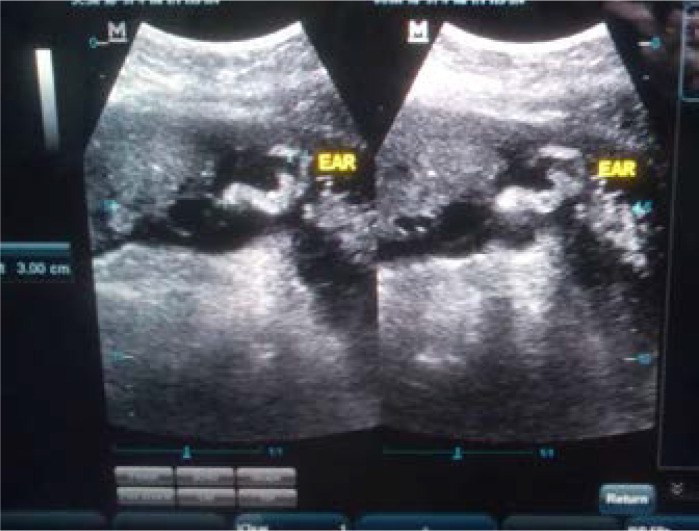
Fetal ear length (calipers) at 36 weeks of gestation in parasagittal views respectively.

With the patient in a supine position, trans-abdominal longitudinal scan was performed on the subject with 3.5MHz curved linear array transducer. From the sagittal plane of the fetal face, the image of the fetal ear is visualised in parasagittal scan tangential to the calvarium, revealing every contour of the ear. Fetal ear length was obtained, with the help of the electronic caliper, in both coronal and parasagittal views, and is defined as the maximal distance from the apical part of the helix to the caudal part of the ear lobe (Fig. 1 and 2). Three measurements were obtained and the average entered as the ear length for the gestational age of the fetus. The average ear length for the corresponding gestational age was used to create a baseline reference value for the population studied. In assessment of the fetal ear, the right or the left ear was chosen according to which was most easily visible during the examination^1^, ^4^. In addition to the standard fetal biometry measurements (BPD, HC, AC and FL), a complete anomaly ultrasound scan was done in each case.

Intra- and inter-observer variability was assessed prior to the commencement of data collection to ensure reliability of measurements. Thirty-two that met the inclusion criteria were selected to assess the reliability of ear length measurement technique. Two sonographers with more than ten years' experience in obstetric sonography independently obtained two measurements of fetal ear length. Each sonographer obtained the measurements at least ten minutes apart to reduce bias. The sonographers were blinded to their measurements and to each other's measurements. Blinding was achieved by masking the read out portion of the ultrasound monitor with a black cellophane tape. Coefficient of variation (COV) for duplicate measurements for intra-observer measurements and inter-observer measurements were obtained. The COVs were less than 5% for intra-observer and less than 15% for inter-observer measurements and both indicate good reproducibility of measurement.

Statistical analysis was performed using Statistical Package for Social Sciences (SPSS) version 20 (IBM Corp. Armonk, NY: USA). The patients were divided into groups according to gestational age of the fetus. The relationship between gestational age in weeks and fetal ear length in millimetres was analysed using simple linear regression. A regression model was constructed for ear length, with the best adjustments represented by a formula. The normal ranges/values of fetal ear length in millimeter were expressed as the median, 5th, 25th, 75th and 95^th^ percentiles. Pearson correlation coefficient (r) and linear regression equation were used to assess strength of correlations (or level of association) and linear relationship between fetal ear length and gestational age, maternal age and biometric parameters (BPD, FL, AC, HC). A p<0.05 was considered statistically significant.

## Results

Table 1 showed the mean and standard deviation distribution, as well as the median and range, of the parameters studied in the 551 study participants. The mean + SD of the maternal age, gestational age, fetal ear length, Femural length,, Biparietal Diameter, Abdominal Circumference, and Head Circumference were (29.01±4.88yrs), (30.57±5.78wks), (23.77±6.36mm), (57.81±13.26mm), (75.50±14.72mm),(266.90±115.77 mm) and (275.20±52.27mm) respectively.

**Table 1:** Descriptive characteristic of studied parameters in the participants (N= 551)

Parameters	Means(SD)	Median	Range (min-max)
**Maternal Age(yrs)**	29.01(4.88)	29.0	18–43
**Gestational age by Last menstrual** **period(weeks)**	30.57(5.78)	32.0	17–41
**Fetal Ear length(mm)**	23.77(6.36)	24.0	7.30–75.60
**Femural Length(mm)**	57.81(13.26)	60.7	22.6–83.7
**Biparietal Diameter(mm)**	75.50(14.72)	79.1	35.6–99.2
**Abdominal Circumference(mm)**	266.90(115.77)	271.0	108–2552
**Head Circumfernce(mm)**	275.20(52.27)	289.0	78.7–365.0

**Figure 3:**
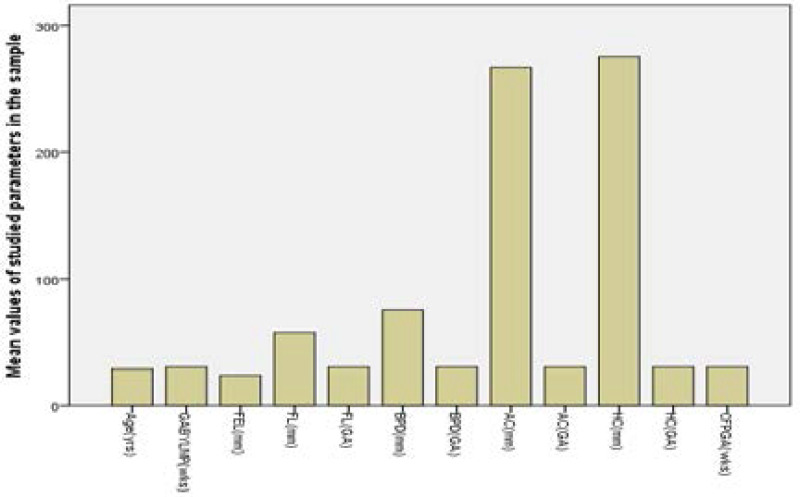
Graphic presentation of the mean distribution of parameters in the studied population

Figure 3 shows graphic presentation of the mean distribution of parameters in the studied sample of the pregnant women. The GA from all the parameters including fetal ear length appeared comparable.

**Figure 4:**
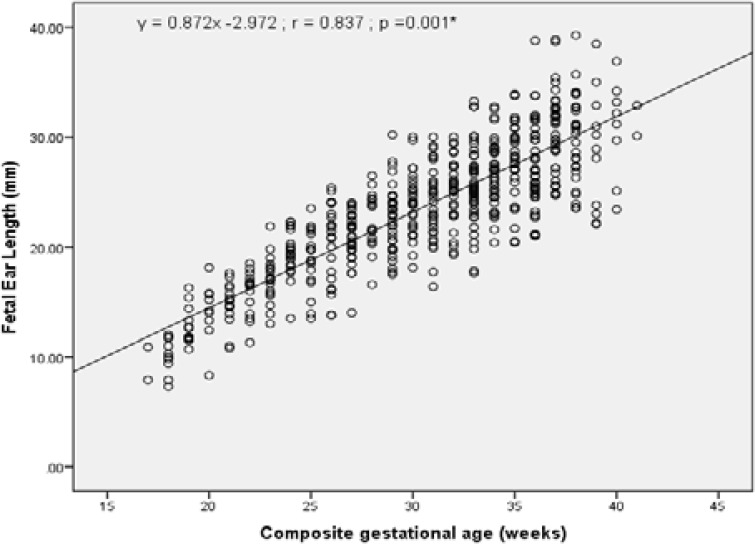
Correlation between FEL and Composite gestational age

A baseline reference value was developed for fetal ear length measurement against gestational ages of 17weeks to 41weeks and presented in Table 2. The median (50th percentile) of FEL measurements increased from 9.40 mm in the 17 weeks of GA to 31.50 mm at 41weeks of GA. The 50^th^ percentile mean baseline value for fetal ear length at 24 weeks gestation was 19.55mm. Thus, in relation to gestational age, the normal reference at 50^th^ percentile range for FEL was 9.40–31.50mm. The median (50th percentile) and the 95^th^ percentile of FEL were 24.00 mm and 32.80 mm, respectively for the studied population regardless of GA. Thus, the reference 95^th^ percentile mean baseline value for FEL was 32.80 mm. This suggests therefore that the normal reference values of FEL among pregnant women between 17–41 weeks of gestation in the studied population was 13.26–32.80. The relationship between fetal ear length and gestational age as shown in Figure 4 appears to be linear (Fetal ear length [millimeters] = 0.872*Gestational age [weeks] – 2.972), with an r value (r = 0.837; p = 0.001).

**Table 2:** The baseline values for fetal ear length among pregnant women between 17 – 41 weeks gestation at each composite gestational age interval expressed in percentile

Gestational age(wks)	Mean(SD)	Fetal ear length percentile by each composite gestational age						
		5	10	25	50	75	90	
**17**	9.40(2.12)	7.90	7.90	7.90	9.40	.	.	.
**18**	10.12(1.60)	7.30	7.36	9.03	10.15	11.73	11.98	.
**19**	12.82(1.72)	10.70	10.94	11.63	12.20	14.13	16.03	.
**20**	14.11(2.74)	8.30	8.30	12.85	14.20	15.75	.	.
**21**	14.79(1.96)	10.80	10.92	13.90	15.10	16.30	17.42	.
**22**	15.76(2.09)	11.30	12.25	13.80	16.55	17.23	18.25	.
**23**	17.42(2.61)	13.00	13.90	15.80	17.10	18.40	21.90	.
**24**	19.41(2.12)	13.67	16.98	18.25	19.35	21.28	22.27	22.30
**25**	18.90(2.72)	13.50	13.90	17.00	19.70	21.20	21.90	.
**26**	20.30(3.60)	13.80	14.37	16.88	21.15	23.25	24.80	25.36
**27**	20.84(2.27)	15.62	17.60	19.73	20.95	22.70	23.72	23.96
**28**	22.39(2.56)	16.60	18.60	20.65	21.70	24.35	25.86	.
**29**	22.55(3.03)	17.65	18.35	19.88	22.85	24.23	27.20	28.40
**30**	23.88(2.90)	18.54	20.16	21.70	24.00	25.70	27.70	29.88
**31**	23.91(3.44)	17.11	19.92	21.38	23.75	26.20	28.93	29.56
**32**	25.11(3.03)	19.40	20.10	22.95	25.50	27.40	29.40	29.75
**33**	25.79(3.77)	18.10	20.61	23.78	25.35	28.33	31.99	32.80
**34**	26.62(2.98)	21.60	22.90	24.50	26.70	28.70	31.60	32.60
**35**	27.75(3.53)	20.46	22.04	25.90	28.00	30.00	31.76	33.84
**36**	27.19(3.97)	21.10	21.65	24.75	26.45	30.13	31.97	33.80
**37**	30.52(3.51)	24.81	25.52	27.95	31.40	32.60	34.86	38.37
**38**	29.85(3.94)	23.55	23.87	27.00	30.50	32.70	34.26	37.68
**39**	28.70(5.51)	22.10	22.12	23.00	28.90	32.90	37.80	.
**40**	30.74(4.55)	23.40	23.40	26.25	31.70	33.95	.	.
**41**	31.50(1.98)	30.10	30.10	30.10	31.50	.	.	.
**CFPGA** **(17–41wks)**	**23.77(6.36)**	**13.26**	**15.70**	**19.80**	**24.00**	**27.80**	**31.68**	**32.80**

To investigate whether there is a correlation between other fetal biometric measurements (BPD, HC, AC and FL) with fetal ear length; the relationship of these biometric measurements (GA) to ear length (mm) was also assessed and is presented in Table 3. The values indicate strong positive correlation with FL, BPD, HC and AC. There was a strong positive correlation between fetal ear length and BPD (r =0.830, R2 = 68.89%); FL (r =0.836, R2 = 69.89%); AC (r =0.823, R2 = 67.73%); and HC (r =0.835, R2 = 69.72%).

**Table 3:** 

Gestational age(wks)	N(%)	Mean(SD)	Fetal ear length percentile by each composite gestational age						
			5	10	25	50	75	90	95
**17**	12(2.2)	9.40(2.12)	7.90	7.90	7.90	9.40	.	.	.
**18**	12(2.2)	10.12(1.60)	7.30	7.36	9.03	10.15	11.73	11.98	.
**19**	12(2.2)	12.82(1.72)	10.70	10.94	11.63	12.20	14.13	16.03	.
**20**	13(2.4)	14.11(2.74)	8.30	8.30	12.85	14.20	15.75	.	.
**21**	15(2.7)	14.79(1.96)	10.80	10.92	13.90	15.10	16.30	17.42	.
**22**	14(2.5)	15.76(2.09)	11.30	12.25	13.80	16.55	17.23	18.25	.
**23**	19(3.4)	17.42(2.61)	13.00	13.90	15.80	17.10	18.40	21.90	.
**24**	20(3.6)	19.41(2.12)	13.67	16.98	18.25	19.35	21.28	22.27	22.30
**25**	19(3.4)	18.90(2.72)	13.50	13.90	17.00	19.70	21.20	21.90	.
**26**	22(4.0)	20.30(3.60)	13.80	14.37	16.88	21.15	23.25	24.80	25.36
**27**	28(5.1)	20.84(2.27)	15.62	17.60	19.73	20.95	22.70	23.72	23.96
**28**	17(3.1)	22.39(2.56)	16.60	18.60	20.65	21.70	24.35	25.86	.
**29**	30(5.4)	22.55(3.03)	17.65	18.35	19.88	22.85	24.23	27.20	28.40
**30**	27(4.9)	23.88(2.90)	18.54	20.16	21.70	24.00	25.70	27.70	29.88
**31**	30(5.4)	23.91(3.44)	17.11	19.92	21.38	23.75	26.20	28.93	29.56
**32**	27(4.9)	25.11(3.03)	19.40	20.10	22.95	25.50	27.40	29.40	29.75
**33**	33(6.0)	25.79(3.77)	18.10	20.61	23.78	25.35	28.33	31.99	32.80
**34**	35(6.3)	26.62(2.98)	21.60	22.90	24.50	26.70	28.70	31.60	32.60
**35**	31(5.6)	27.75(3.53)	20.46	22.04	25.90	28.00	30.00	31.76	33.84
**36**	36(6.5)	27.19(3.97)	21.10	21.65	24.75	26.45	30.13	31.97	33.80
**37**	35(6.3)	30.52(3.51)	24.81	25.52	27.95	31.40	32.60	34.86	38.37
**38**	28(5.1)	29.85(3.94)	23.55	23.87	27.00	30.50	32.70	34.26	37.68
**39**	14(2.5)	28.70(5.51)	22.10	22.12	23.00	28.90	32.90	37.80	.
**40**	12(2.2)	30.74(4.55)	23.40	23.40	26.25	31.70	33.95	.	.
**41**	10(1.8)	31.50(1.98)	30.10	30.10	30.10	31.50		.	.
**CFPGA** **(17–41wks)**	**551(100)**	**23.77(6.36)**	**13.26**	**15.70**	**19.80**	**24.00**	**27.80**	**31.68**	**32.80**

Graphical presentations of the correlation between fetal ear length and other fetal biometric measurements (BPD, HC, AC and FL) are as shown in Figures I – IV under Appendix 1. There was however no strong correlation between fetal ear length and maternal age (See Figure V under Appendix 1).

**Figure I:**
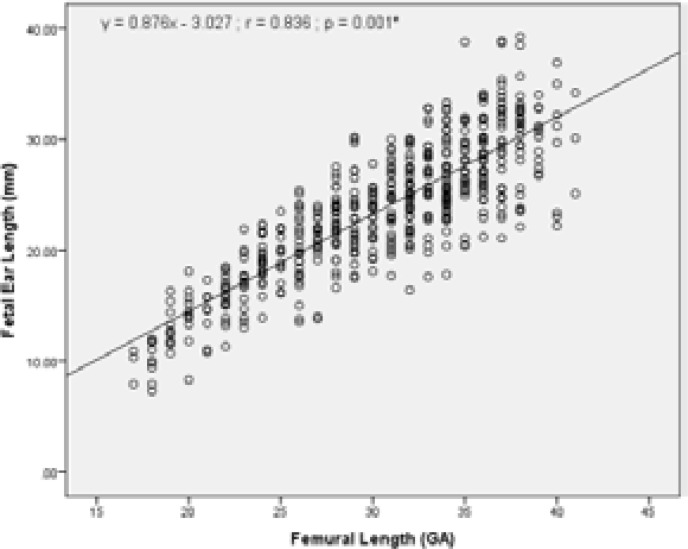
Correlation between FEL and Femural length

**Figure II:**
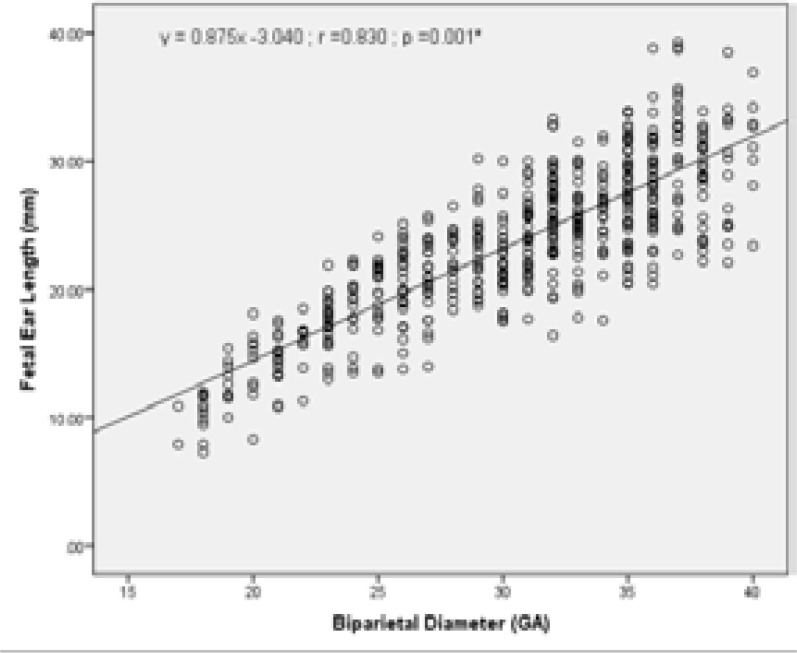
Correlation between FEL and Biparietal Diameter

**Figure III:**
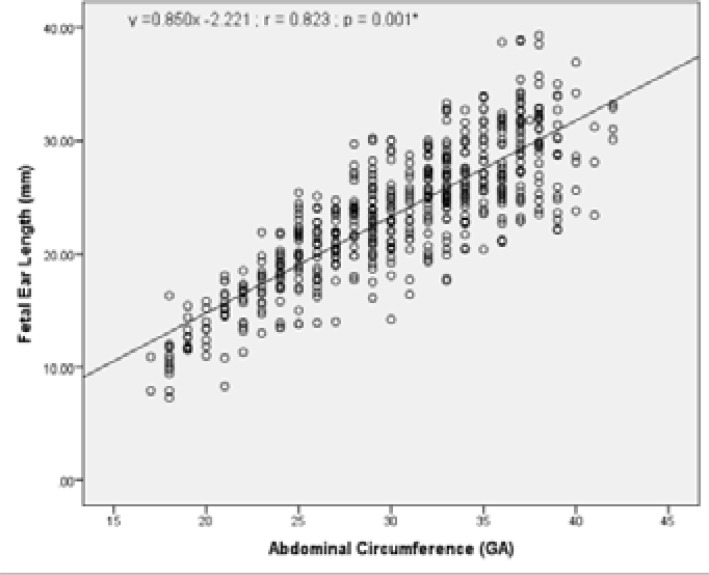
Correlation between FEL and Abdominal circumference

**Figure IV:**
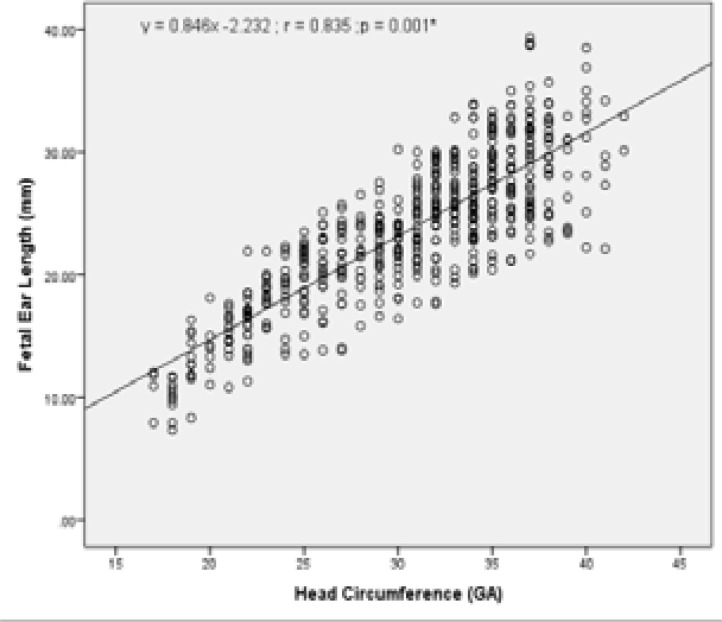
Correlation between FEL and Head circumference

**Figure V:**
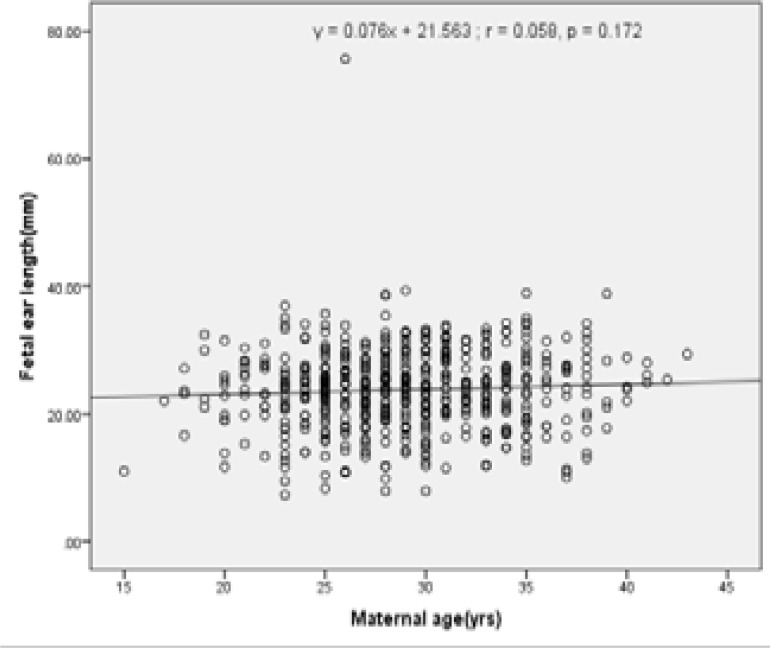
Correlation between FEL and Maternal age

## Discussion

Visualization of the fetal ear is not a part of the routine assessment especially in the developing countries, and hence has received little attention in the ultrasound literatures. Anupama^21^ suggested assessment of the fetal ear when fetal anomalies is suspected especially those with facial anomalies, as this could help in the differential diagnosis.

Abnormal small ear has been noted to be an additional sonographic marker for detection of fetal aneuploidies^20^,^22^. Studies have been done among different ethnic populations like the Turkish, Indians, Nepalese and Americans to establish a nomogram for fetal ear length, to help in the antenatal prediction of chromosomal abnormalities and estimation of gestational age. This study however provided mean baseline reference value for fetal ear length for pregnant women in Port Harcourt, Nigeria.

In this study, the baseline reference value for fetal ear length was generated using gestational age from combined fetal biometric parameters like FL, BPD, AC and HC. The fetal ear length was observed to increase with the gestational age. This is in conformity with previous findings among Turkish^3^, Indians^4^, Americans^20^, Brazilians^23^, Japanese^24^, Americans^25^, ^26^, and British^27^ which found a linear relationship between fetal ear length with increase in gestational age.

The mean±SD fetal ear length increased gradually from 9.40±2.12mm (range = 7.90–10.90mm) at 17 weeks to 31.50±1.98mm (range = 30.10–32.90mm) at 41 weeks. The reference 95th percentile mean baseline value for FEL was 32.80 mm. Thus, at 95% confidence limit, covering about 95% of the FEL measurement, values that one may expect in the studied population will fall within the range: 13.26–32.80 mm. Then 2.5% will be lowerhan the lower limit of 13.26 while the remaining 2.5% will be larger than the upper limit of 32.80.

The reference values from this study were closely related with minimal difference to that reported among Americans by Chitkara et al.^20^ which increased from 8.5mm at 15weeks GA to 32.6mm at 40 weeks GA. These could be because of the similarity in fetal ear length measuring technique, where the average of three measurements for all studied subjects was used. However, it was lower when compared to the nomographic values observed among the Turkish by Ozdemir et al.^3^ and Indians by Rajanna et al.,^4^. The differences could be due to ethnicity/racial variation as revealed by Skaria et al.^28^, who reported that ear size varies according to ethnic group.

The positive correlations of fetal ear length with FL, BPD, AC and HC were also consistent with other studies' finding among the Nepalese^2^, Turkish^3^, Indians^4^,^21^, and Brazilians23. This study showed no significant correlation between fetal ear length and maternal age (MA). This indicates that maternal age does not influence fetal ear length.

There is need to obtain the normal reference values for fetal ear length among pregnant women in Nigerian locality. This study therefore provides normal baseline reference value for fetal ear length in an Igbo subgroup in Port Harcourt, Nigeria. The study also provides linear relationship and positive correlations between fetal ear length and composite gestational age, FL, BPD, AC and HC.

Data presented in this study can be used as an additional sonographic marker to screen fetal aneuploidies between 17–41 weeks GA to further increase the sensitivity of its detection, and the reference values obtained here may be more reliable than using those obtained from other ethnic groups.
